# Topological and Spectral Properties of Wavy Zigzag Nanoribbons

**DOI:** 10.3390/molecules28010152

**Published:** 2022-12-24

**Authors:** Micheal Arockiaraj, J. Celin Fiona, S. Ruth Julie Kavitha, Arul Jeya Shalini, Krishnan Balasubramanian

**Affiliations:** 1 Department of Mathematics, Loyola College, Chennai 600034, India; marockiaraj@gmail.com (M.A.); celinfiona99@gmail.com (J.C.F.); juliesuresh91@gmail.com (S.R.J.K.); 2 Department of Mathematics, Women’s Christian College, Chennai 600006, India; aruljeyashalini@gmail.com; 3 School of Molecular Sciences, Arizona State University, Tempe, AZ 85287-1604, USA

**Keywords:** graphene nanoribbons, topological indices, edge partition, spectral properties, HOMO-LUMO gaps

## Abstract

Low-dimensional graphene-based nanomaterials are interesting due to their cutting-edge electronic and magnetic properties. Their large surface area, strong mechanical resistance, and electronic properties have enabled potential pharmaceutical and opto-electronic applications. Graphene nanoribbons (GNRs) are graphene strips of nanometer size possessing zigzag and armchair edge geometries with tunable widths. Despite the recent developments in the characterization, design and synthesis of GNRs, the study of electronic, magnetic and topological properties, GNRs continue to pose a challenge owing to their multidimensionality. In this study, we obtain the topological and electronic properties of a series of wave-like nanoribbons comprising nanographene units with zigzag-shaped edges. The edge partition techniques based on the convex components are employed to compute the mathematical formulae of molecular descriptors for the wave-like zigzag GNRs. We have also obtained the spectral and energetic properties including HOMO-LUMO gaps, bond delocalization energies, resonance energies, C13 NMR and ESR patterns for the GNRs. All of these computations reveal zero to very low HOMO-LUMO gaps that make these nanoribbons potential candidates for topological spintronics.

## 1. Introduction

Polynuclear/polycyclic aromatic hydrocarbons (PNA/PCAH) and their heterocyclic derivatives are of considerable interest in environmental pollution, carcinogenicity, and other potential toxicological applications pertinent to public health [1,2,3,4]. However, the 2D materials derived from these compounds exhibit distinct electronic properties, including their macrocyclic conjugation, making them attractive candidates for devices such as organic semiconductors, solar cells, and transistors [5,6,7], and hence they are of interest in opto-electronics, photographic products, pharmaceuticals, functional plastics, and liquid crystals [7]. Henceforth, these polycyclic compounds have been the subject of experimental and theoretical investigations over the past decades [7,8,9,10]. Graphene, a two-dimensional PCAH derivative, possesses high strength, supreme thermal conductivity, gas impermeability, giant charge carrier mobility, and the ability to transport without scattering even at ambient temperatures [11,12,13,14,15,16]. The rate of electron transfer of around $10^{3}$ m/s through the π−π bond and the structural configuration of graphene contribute to electrical and mechanical properties that make them suitable as biological sensors [17]. Its low weight, outstanding robustness, high conductivity, and chemical stability, make it suitable as an anode material in lithium-ion batteries [18], whereas its nanoelectronic conductance and ambipolar behavior play a vital role in graphene-based field effect transistors, in the fabrication of ion exchange membranes, and in several other chemical and biological applications [19,20,21,22,23,24,25,26].

Graphene nanoribbons are unzipped, segmented, quasi-one-dimensional, planar, and elongated thin strips of graphene sheets with repeated hexagonal components of carbon configured in a nanoscale dimension [27]. On the basis of their edge symmetries and structures, the edge sets of nanoribbons are categorized as armchair, zigzag or intermediate edge characters [28,29], and these distinct edge classes are observed to have a strong influence on the electronic ground state and reactivity of the material [30]. For instance, non-zero bandgaps of GNRs with hydrogen passivation arise from quantum confinement of edges for zigzag ribbon whereas from staggered sublattice potential owing to edge magnetization for the armchair type edges [31,32,33]. Furthermore, it is possible to realize GNRs of varied widths through patterning epitaxial graphenes [34,35,36], which thus provides control of their structural geometry, making them a potential candidate for the development of new nanoelectronic devices [37,38]. GNR derivatives such as graphene-oxide nanoribbons (GONRs) have been discovered to have effective functionalities in biomedical applications, including anti-cancer and gene therapies [39]. The applicability of GNRs and their derivatives as biomedical and electronic materials is thus a growing research area and these structures still require further studies in order to gain insights into their properties as a function of their structures.

**Figure 1:**
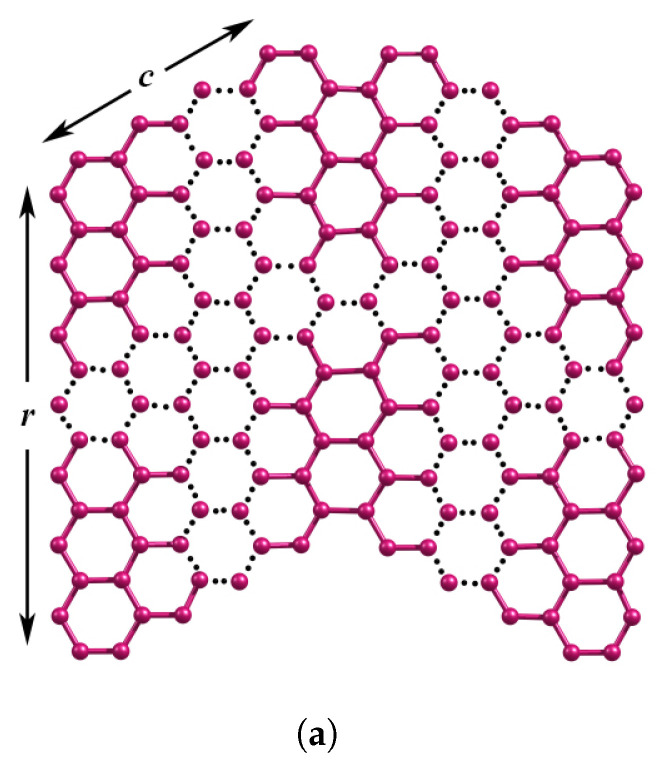

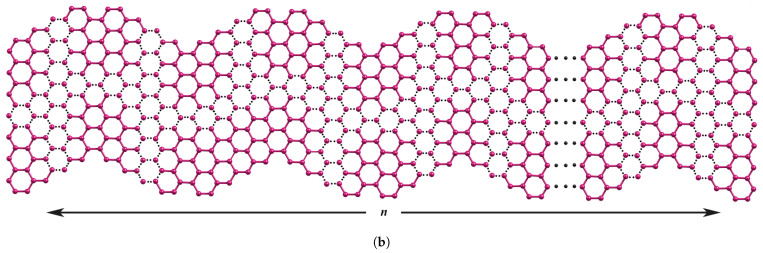
(**a**) Zigzag edges of nanographene ZNR (r,c) (**b**) *n*-wave series of WNR (r,c,n) .

Stimulated by several ongoing experimental studies on GNRs, in the present study we have investigated topological, spectral and energetic studies to characterize a series of graphene nanoribbons that are employed in designing the magnetic topological graphene-based nanoribbons (MT-GNRs) with non-trivial topology [40]. The wave-like zigzag series WNR (r,c,n) considered in this study is obtained by chaining *n* units of the trans-coupled zigzag-edged nanographene unit ZNR (r,c) , where ZNR (r,c) consists of two copies of parallelogram-type benzenoid systems [41], as shown in Figure 1. As physico-chemical properties of these materials are correlated with the underlying molecular structure [42,43,44,45], there is a clear and compelling need to characterize the underlying topological connectivity of GNRs as a function of their width and length. Topological descriptors are structure-based numeric entities that capture the underlying network connectivity through graph theory. These techniques together with energy spectra, delocalization energies, bond resonance energies, aromatic sextets, etc., provide considerable insight into their stabilities, reactivities, aromaticity, ring currents, and so forth [46,47,48,49,50,51,52,53]. As these topological descriptors are invariant to labelings, they play a vital role in the quantitative analysis of structural activity, property, and toxicity relationships (QSAR/QSPR/QSTR) [54]. A wide range of topological descriptors have been developed to date for the characterization of chemical structures and nanomaterials [43,55,56,57,58,59,60,61,62,63,64,65].

In this paper, we compute two different classes of topological descriptors for wave-like graphene-based nanoribbons. Furthermore we employ graph theory based techniques for machine learning of C13 NMR and ESR spectral patterns of the GNRs. We also compute their HOMO-LUMO gaps, bond delocalization energies, total π -electronic energies and resonance energies/bond as a function of lengths and widths to gain insights into their stabilities and reactivities as a function of their ribbon widths and lengths.

## 2. Computational Techniques

The computation of topological descriptors can theoretically provide quantitative relationships to the physico-chemical properties of chemical structures under study. The technique for such computation is predominately based on the classification of chemical structures under the subclass of hypercubes. A hypercube $\left(Q_{n}\right)$ is an undirected graph of dimension *n* consisting of $2^{n}$ vertices labelled from 0 to $2^{n}-1$ and edges connecting any two vertices if and only if their binary representations differs exactly by one bit. Partial cubes are isometric subgraphs of hypercubes. Many chemical graphs such has trees, phenylenes, and benzenoid graphs are comes under partial cube family. In our research, we considered the partial cube family $P_{Q}$ with a class $W_{Q}$ having $V(W_{Q})$ and $E(W_{Q})$ respectively as vertex and the edge sets.

The characterization of $W_{Q}$ due to Djokovi $\overset{´}{c}$ −Winkler was examined on the edges of $W_{Q}$ using a special relation known as Θ , where Θ defines that two edges $p_{i}=r_{i}s_{i}$ and $q_{j}=r_{j}s_{j}$ are Θ -related if $d_{W_{Q}}\left(r_{i},r_{j}\right)+d_{W_{Q}}\left(s_{i},s_{j}\right)$ ≠ $d_{W_{Q}}\left(r_{i},s_{j}\right)$ + $d_{W_{Q}}\left(r_{j},s_{i}\right)$ , where $d_{W_{Q}}\left(r_{i},s_{j}\right)$ represents the distance between $r_{i}$ and $s_{j}$ . As we can see, the relation Θ is equivalence in $W_{Q}$ , the different classes of $W_{Q}$ denoted by $B_{1},\ldots ,B_{k}$ [66,67]. Thus, the class $B_{i}$ , 1≤i≤k , splits $W_{Q}$ into 2-components $\left\{W_{Q}^{1}\left(B_{i}\right),W_{Q}^{2}\left(B_{i}\right)\right\}$ graph. Let $n_{1}\left(B_{i}\right)=\left|V\left(W_{Q}^{1}\left(B_{i}\right)\right)\right|$ , $n_{2}\left(B_{i}\right)=\left|V\left(W_{Q}^{2}\left(B_{i}\right)\right)\right|$ , $m_{1}\left(B_{i}\right)=\left|E\left(W_{Q}^{1}\left(B_{i}\right)\right)\right|$ and $m_{2}\left(B_{i}\right)=\left|E\left(W_{Q}^{2}\left(B_{i}\right)\right)\right|$ . Consequently, we have $n_{1}\left(B_{i}\right)+n_{2}\left(B_{i}\right)=\left|V\left(W_{Q}\right)\right|$ and $m_{1}\left(B_{i}\right)+m_{2}\left(B_{i}\right)=|E\left(W_{Q}\right)\left|-\right|B_{i}|$ .

The distance-based topological descriptors are derived from the above-defined Θ -classes and their component parameters, which are formally given below.

1. Vertex Wiener $W(W_{Q})$ = $\sum_{i=1}^{k}n_{1}\left(B_{i}\right)$ $n_{2}\left(B_{i}\right)$
2. Edge Wiener $W_{e}\left(W_{Q}\right)=\sum_{i=1}^{k}m_{1}\left(B_{i}\right)$ $m_{2}\left(B_{i}\right)$
3. Vertex-edge Wiener $W_{ev}\left(W_{Q}\right)=\frac{1}{2}\sum_{i=1}^{k}[n_{1}\left(B_{i}\right)$ $m_{2}\left(B_{i}\right)+n_{2}\left(B_{i}\right)$ $m_{1}\left(B_{i}\right)]$
4. Vertex Szeged $Sz_{v}\left(W_{Q}\right)$ = $\sum_{i=1}^{k}$ $|B_{i}|$ $n_{1}\left(B_{i}\right)$ $n_{2}\left(B_{i}\right)$
5. Edge Szeged $Sz_{e}\left(W_{Q}\right)$ = $\sum_{i=1}^{k}$ $|B_{i}|$ $m_{1}\left(B_{i}\right)$ $m_{2}\left(B_{i}\right)$
6. Edge-vertex Szeged $Sz_{ev}\left(W_{Q}\right)$ = $\frac{1}{2}\sum_{i=1}^{k}\left|B_{i}\right|$ $[n_{1}\left(B_{i}\right)$ $m_{2}\left(B_{i}\right)+n_{2}\left(B_{i}\right)$ $m_{1}B_{i})]$
7. Padmakar-Ivan $PI\left(W_{Q}\right)=|E\left(W_{Q}\right){|}^{2}-\sum_{i=1}^{k}{\left|B_{i}\right|}^{2}$
8. Schultz $S\left(W_{Q}\right)=2\sum_{i=1}^{k}[n_{1}\left(B_{i}\right)$ $m_{2}\left(B_{i}\right)+n_{2}\left(B_{i}\right)$ $m_{1}\left(B_{i}\right)]+|E\left(W_{Q}\right)||V\left(W_{Q}\right)|$
9. Gutman $Gut\left(W_{Q}\right)=\sum_{i=1}^{k}[4m_{1}\left(B_{i}\right)$ $m_{2}\left(B_{i}\right)-|B_{i}{|}^{2}]+2|E\left(W_{Q}\right){|}^{2}$
10. Vertex Mostar $Mo_{v}\left(W_{Q}\right)$ = $\sum_{i=1}^{k}$ $|B_{i}|$ $|n_{1}\left(B_{i}\right)-n_{2}\left(B_{i}\right)|$
11. Edge Mostar $Mo_{e}\left(W_{Q}\right)$ = $\sum_{i=1}^{k}$ $|B_{i}|$ $|m_{1}\left(B_{i}\right)-m_{2}\left(B_{i}\right)|$

Since the computation of degree-based indices required edge classification of $E(W_{Q})$ , we denote $d_{p_{j}}$ to represent the degree of $p_{j}$ that counts the edges that are incident to $p_{j}$ . A collection of vertices that are adjacent to $p_{j}$ are referred to as the neighbors of $p_{j}$ and are represented by $N_{p_{j}}$ . The sum of degrees to the neighbors of $p_{j}$ is denoted by $s_{p_{j}}$ and computed by $s_{p_{j}}=\sum_{x_{i}\in N_{p_{j}}}d_{x_{i}}$ . It is clear that $1\leq d_{p_{j}}\leq \left|V\left(W_{Q}\right)\right|-1$ and $2\leq s_{p_{j}}\leq (|V\left(W_{Q}\right){\left|-1\right)}^{2}$ . Let $I=\{\left(s,t\right):s\leq t,1\leq t\leq (|V\left(W_{Q}\right){\left|-1\right)}^{2}\}$ .

Let $d_{st}\left(W_{Q}\right)=|\{w_{j}u_{j}\in E\left(W_{Q}\right)\;:\;d_{w_{j}}=s\;$ and $\;d_{u_{j}}=t\}|$ and $s_{st}\left(W_{Q}\right)=|\{w_{j}u_{j}\in E\left(W_{Q}\right)\;:\;s_{w_{j}}=s\;$ and $\;s_{u_{j}}=t\}|$ . For #∈{d,s} , we denote $\psi^{\#}\left(W_{Q}\right)=\sum_{(s,t)\in I}{\#}_{st}\left(W_{Q}\right)\;\psi \left(s,t\right)$ to represent the degree/degree sum based indices [68,69,70], where ψ(s,t) defined for the first Zagreb index $M_{1}\left(s,t\right)=s+t$ , Sombor index $SO\left(s,t\right)=\sqrt{s^{2}+t^{2}}$ , second Zagreb index $M_{2}\left(s,t\right)=st$ , sum-connectivity index $SC\left(s,t\right)=\frac{1}{\sqrt{s+t}}$ , geometric-arithmetic index $GA\left(s,t\right)=\frac{2\sqrt{st}}{s+t}$ , augmented Zagreb index $AZ\left(s,t\right)={\left(\frac{st}{s+t-2}\right)}^{3}$ , inverse sum indeg index $ISI\left(s,t\right)=\frac{st}{s+t}$ , atom-bond connectivity index $ABC\left(s,t\right)=\sqrt{\frac{s+t-2}{st}}$ , forgotten index $F\left(s,t\right)=s^{2}+t^{2}$ , symmetric degree division index SDD = $\frac{s}{t}+\frac{t}{s}$ , and harmonic index $H\left(s,t\right)=\frac{2}{s+t}$ .

## 3. Results and Discussion

As the wave-like nanoribbons are the most challenging material for the forthcoming generation, we now investigate the topological characteristics in this section.

### 3.1. Distance Based Descriptors

The wave series of WNR (r,c,n) is composed of *n*-units of trans-coupled zigzag-edged nanographene units ZNR (r,c) , where each ZNR (r,c) contains 4(cr+c+r) vertices and 2c(3r+2)+5r−2 edges, and thus WNR (r,c,n) has vertices and edges as 4n(cr+c+r) and n(2c(3r+2)+5r−2)+r(n−1) respectively. Since the topological properties of WNR (r,c,n) behave differently for the cases r≤c and r>c , we first considered the case r≤c . In this section, we use $\psi (G/B_{i})$ to denote the quantitative values emerging from the components with respect to the class $B_{i}$ of Θ -relation for the index function ψ in which $\psi \in \{W,W_{e},W_{ev},Sz_{v},Sz_{e},Sz_{ev},PI,S,Gut,Mo_{v},Mo_{e}\}$ .

**Theorem** **1.** *Let G be a wave-like nanoribbons* WNR (r,c,n) *, where r≤c , n≥1 , and r≥2 .*

*1.*  *
$\begin{array}{l} W(G)=\frac{2}{15}(r^{4}\left(15n-5c+15cn-10\right)-r^{5}+r^{3}\left(30n-20c+80cn+10c^{2}n\right)+r^{2}(80c^{3}n^{3}+240c^{2}n^{3}+30c^{2}n+240cn^{3}+10cn+5c+80n^{3}-80n+\\ 40)+r\left(160c^{3}n^{3}+320c^{2}n^{3}+160cn^{3}-70cn+20c+30n-29\right)+80c^{3}n^{3}+80c^{2}n^{3}-20c^{2}n-5cn) \end{array}$
*
*2.*  *
$\begin{array}{l} W_{e}\left(G\right)=\frac{1}{30}(r^{4}\left(135n-45c+135cn-90\right)-9r^{5}+r^{3}\left(90n-150c+540cn+90c^{2}n+65\right)+r^{2}(720c^{3}n^{3}+2160c^{2}n^{3}-900c^{2}n^{2}+180c^{2}n+2160cn^{3}-\\ 1800cn^{2}-135cn+165c+720n^{3}-900n^{2}-495n+300)+r(960c^{3}n^{3}+1440c^{2}n^{3}-960c^{2}n^{2}-60c^{2}n-480cn^{2}+240cn-30c-480n^{3}+480n^{2}+\\ 960n-596)+320c^{3}n^{3}-240c^{2}n^{2}-120c^{2}n-240cn^{3}+240cn^{2}+40cn+60c+80n^{3}-60n^{2}-530n+360) \end{array}$
*
*3.*  *
$\begin{array}{l} W_{ev}\left(G\right)=\frac{1}{30}(r^{4}\left(90n-30c+90cn-60\right)-6r^{5}+r^{3}\left(120n-110c+420cn+60c^{2}n+20\right)+r^{2}(480c^{3}n^{3}+1440c^{2}n^{3}-300c^{2}n^{2}+150c^{2}n+1440cn^{3}-\\ 600cn^{2}-90cn+75c+480n^{3}-300n^{2}-480n+240)+r(800c^{3}n^{3}+1440c^{2}n^{3}-420c^{2}n^{2}-30c^{2}n+480cn^{3}-360cn^{2}-200cn+65c-160n^{3}+60n^{2}+\\ 430n-284)+320c^{3}n^{3}+160c^{2}n^{3}-120c^{2}n^{2}-100c^{2}n-160cn^{3}+60cn^{2}+50cn-120n+90) \end{array}$
*
*4.*  *
$\begin{array}{l} Sz_{v}\left(G\right)=\frac{1}{3}(r^{4}\left(6n-3c+6cn-7\right)+r^{3}\left(48c^{3}n^{3}+144c^{2}n^{3}+144cn^{3}+28cn-22c+48n^{3}+4n-8\right)+r^{2}(128c^{3}n^{3}+4c^{3}n+240c^{2}n^{3}+36c^{2}n-12c^{2}+\\ 96cn^{3}+48cn-16n^{3}-8n+31)+r\left(112c^{3}n^{3}+4c^{3}n+80c^{2}n^{3}-8c^{2}n+12c^{2}-32cn^{3}-70cn+49c-52n+26\right)+32c^{3}n^{3}-16c^{2}n^{3}+4c^{2}n+22cn-\\ 24c+48n-42) \end{array}$
*
*5.*  *
$\begin{array}{l} Sz_{e}\left(G\right)=\frac{1}{30}(r^{4}\left(195n-60c+195cn-160\right)-3r^{5}+r^{3}(1080c^{3}n^{3}+3240c^{2}n^{3}-1260c^{2}n^{2}+210c^{2}n+3240cn^{3}-2520cn^{2}+1600cn-440c+1080n^{3}-\\ 1260n^{2}+850n-235)+r^{2}(2160c^{3}n^{3}-180c^{3}n^{2}+60c^{3}n+3240c^{2}n^{3}-2820c^{2}n^{2}+990c^{2}n-240c^{2}-2220cn^{2}+1065cn-60c-1080n^{3}+420n^{2}-\\ 885n+580)+r(1440c^{3}n^{3}-120c^{3}n^{2}+80c^{3}n-780c^{2}n^{2}-390c^{2}n+180c^{2}-1080cn^{3}+1860cn^{2}-2160cn+980c+360n^{3}+900n^{2}-820n+448)+\\ 320c^{3}n^{3}-480c^{2}n^{3}+240c^{2}n^{2}-360c^{2}n+60c^{2}+240cn^{3}+480cn^{2}+40cn-300c-40n^{3}-300n^{2}+340n-360) \end{array}$
*
*6.*  *
$\begin{array}{l} Sz_{ev}\left(G\right)=\frac{1}{60}(r^{4}\left(220n-85c+220cn-200\right)-2r^{5}+r^{3}(1440c^{3}n^{3}+4320c^{2}n^{3}-840c^{2}n^{2}+140c^{2}n+4320cn^{3}-1680cn^{2}+1340cn-580c+1440n^{3}-\\ 840n^{2}+480n-170)+r^{2}(3360c^{3}n^{3}-120c^{3}n^{2}+100c^{3}n+5760c^{2}n^{3}-2160c^{2}n^{2}+1200c^{2}n-330c^{2}+1440cn^{3}-2040cn^{2}+1240cn+55c-960n^{3}-\\ 640n+800)+r(2560c^{3}n^{3}-120c^{3}n^{2}+100c^{3}n+960c^{2}n^{3}-960c^{2}n^{2}-440c^{2}n+330c^{2}-1440cn^{3}+840cn^{2}-2680cn+1330c+160n^{3}+600n^{2}-\\ 1300n+592)+640c^{3}n^{3}-640c^{2}n^{3}+240c^{2}n^{2}-200c^{2}n+160cn^{3}+600cn^{2}+460cn-720c+1080n-1020) \end{array}$
*
*7.*  *
$\begin{array}{l} PI(G)=\frac{2}{3}(r^{3}+r^{2}\left(54c^{2}n^{2}+108cn^{2}-30cn+3c+54n^{2}-30n+9\right)+r\left(72c^{2}n^{2}-3c^{2}n+36cn^{2}-36cn+3c-36n^{2}+3n-7\right)+24c^{2}n^{2}-24cn^{2}+\\ 6cn-6c+6n^{2}+15n-6) \end{array}$
*
*8.*  *
$\begin{array}{l} S(G)=\frac{2}{15}(r^{4}\left(90n-30c+90cn-60\right)-6r^{5}+r^{3}\left(120n-110c+420cn+60c^{2}n+20\right)+r^{2}(480c^{3}n^{3}+1440c^{2}n^{3}-120c^{2}n^{2}+150c^{2}n+1440cn^{3}-\\ 240cn^{2}-120cn+75c+480n^{3}-120n^{2}-510n+240)+r(800c^{3}n^{3}+1440c^{2}n^{3}-120c^{2}n^{2}-30c^{2}n+480cn^{3}-120cn^{2}-230cn+65c-160n^{3}+\\ 430n-284)+320c^{3}n^{3}+160c^{2}n^{3}-100c^{2}n-160cn^{3}+50cn-120n+90) \end{array}$
*
*9.*  *
$\begin{array}{l} Gut(G)=\frac{1}{15}(r^{4}\left(270n-90c+270cn-180\right)-18r^{5}+r^{3}\left(180n-300c+1080cn+180c^{2}n+140\right)+r^{2}(1440c^{3}n^{3}+4320c^{2}n^{3}-720c^{2}n^{2}+360c^{2}n+\\ 4320cn^{3}-1440cn^{2}-750cn+360c+1440n^{3}-720n^{2}-1470n+705)+r(1920c^{3}n^{3}+2880c^{2}n^{3}-480c^{2}n^{2}-150c^{2}n-240cn^{2}-30c-960n^{3}+\\ 240n^{2}+2010n-1262)+640c^{3}n^{3}-240c^{2}n-480cn^{3}+140cn+60c+160n^{3}-910n+660) \end{array}$
*
*10.*  *
$\begin{array}{l} Mo_{v}\left(G\right)=r^{2}\left(12c^{2}n^{2}+24cn^{2}-4cn+6c+12n^{2}-4n+6\right)-2r^{4}+r(20c^{2}n^{2}+16cn^{2}-4cn-10c-4n^{2}-8)+8c^{2}n^{2}-8c^{2}-4cn^{2}-16c-4+\\ 2\left(1-{\left(-1\right)}^{n}\right)(r+c+1)\left|r^{2}-2c-2\right|+2\left(1+{\left(-1\right)}^{n}\right)\left(r+c+1\right)\left|2c-r^{2}+2\right| \end{array}$
*
*11.*  *
$\begin{array}{l} Mo_{e}\left(G\right)=\frac{1}{6}(4r^{3}-18r^{4}+r^{2}(108c^{2}n^{2}+216cn^{2}-36cn+48c+108n^{2}-36n+42)+r\left(144c^{2}n^{2}+72cn^{2}-24cn-84c-72n^{2}+12n-40\right)+\\ 48c^{2}n^{2}-60c^{2}-48cn^{2}-120c+12n^{2}-60)+\left(1-{\left(-1\right)}^{n}\right)\left(r+c+1\right)\left|3r^{2}-r-5c-5\right|+\left(1+{\left(-1\right)}^{n}\right)\left(r+c+1\right)|r-3r^{2}+5c+5| \end{array}$
*

**Figure 2:**
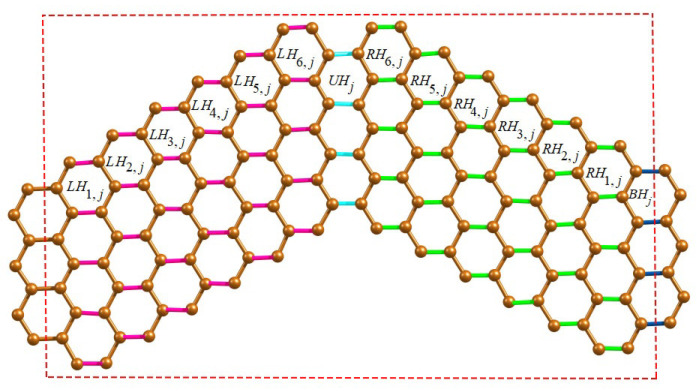
H-type Θ -classes for the *j*th unit of WNR (4,6,n) .

**Proof.**  We first present the Θ -classes of wave-like nanoribbons WNR (r,c,n) and their associated component parameters. Since WNR (r,c,n) is a linear arrangement of *n* units of ZNR (r,c) , we now classify various Θ -classes arising from the jth unit of ZNR (r,c) where 1≤j≤n . We now consider the horizontal edges (H-type) of WNR (r,c,n) and group them into four categories of Θ -classes. The first category of H-type Θ -classes starting from left to right of ZNR (r,c) denoted by $\left\{LH_{i,j}:1\leq i\leq c;1\leq j\leq n\right\}$ , while the second category $\left\{RH_{i,j}:1\leq i\leq c;1\leq j\leq n\right\}$ runs from right to left which is the mirror image of first category. The third and fourth categories of H-type Θ -classes are denoted by $\left\{UH_{j}:1\leq j\leq n\right\}$ , $\left\{BH_{j}:1\leq j\leq n-1\right\}$ respectively, which consists of coupling edges in the upper and bottom wave series. The H-type Θ -classes are shown in Figure 2 and graph parameters are given in Table 1.

**Figure 3:**
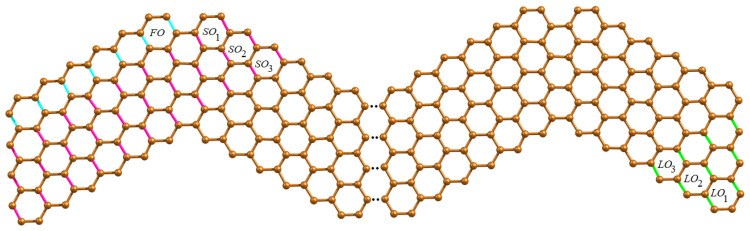
O-type Θ -classes on the initial and final unit of WNR (4,6,n) .

**Figure 4:**
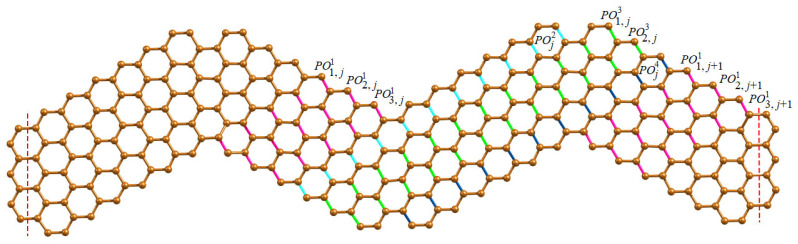
O-type parallelogram Θ -classes on two consecutive units of WNR (4,6,n) .

The obtuse edges (O-type) of WNR (r,c,n) are now taken into consideration, and they are divided into seven categories of Θ -classes. The first category of O-type Θ -class consists of a unique FO class composing of obtuse edges in the top-left corner. Then, we have |FO| = c+1 , $n_{1}\left(FO\right)$ = 2c+1 and $m_{1}\left(FO\right)$ = 2c . The second category of O-type Θ -classes follow the FO class which are denoted by $\left\{SO_{i}:1\leq i\leq r-1\right\}$ as drawn in Figure 3. The third to sixth categories of O-type Θ -classes covering the edges in the lower area of wave series under parallelogram shapes, which are denoted by $\left\{PO_{i,j}^{1}:1\leq i\leq c-r+1;1\leq j\leq n\right\}$ , $\left\{PO_{j}^{2}:1\leq j\leq n-1\right\}$ , $\left\{PO_{i,j}^{3}:1\leq i\leq r-2;1\leq j\leq n-1\right\}$ and $\left\{PO_{j}^{4}:1\leq j\leq n-1\right\}$ respectively. These Θ -classes are shown in Figure 4.

Finally, we define the seventh category of O-type Θ -classes $\left\{LO_{i}:1\leq i\leq r-1\right\}$ to be covered on the last unit of WNR (r,c,n) , as illustrated in Figure 3. The graph parameters for O-type Θ classes are given in Table 2.

The acute edges (A-type) consist of Θ -classes $FA,SA_{i},PA_{i,j}^{1},PA_{j}^{2},PA_{i,j}^{3},PA_{j}^{4}$ and $LA_{i}$ which have symmetry with respect to O-type Θ -classes $FO,SO_{i},PO_{i,j}^{1},PO_{j}^{2},PO_{i,j}^{3},PO_{j}^{4}$ and $LO_{i}$ . Thus, for computing the topological descriptors in the case of A-type classes, we count the numerical quantities of O-type classes twice. Therefore, we obtain the topological expressions by the graph theoretical parameters given in Table 1 and Table 2 using the equation given below.

$$\begin{array}{cc} \psi (G)= & \sum_{j=1}^{n}\left\{\sum_{i=1}^{c}\left(\psi \left(G/FH_{i,j}\right)+\psi \left(G/RH_{i,j}\right)\right)+\psi \left(G/UH_{j}\right)\right\}+\sum_{j=1}^{n-1}\psi \left(G/BH_{j}\right)\\ & +2[\psi \left(G/FO\right)+\sum_{i=1}^{r-1}\psi \left(G/SO_{i}\right)+\sum_{j=1}^{n}\sum_{i=1}^{c-r+1}\psi \left(G/PO_{i,j}^{1}\right)+\sum_{j=1}^{n-1}\psi \left(G/PO_{j}^{2}\right)\\ & +\sum_{j=1}^{n-1}\sum_{i=1}^{r-2}\psi \left(G/PO_{i,j}^{3}\right)+\sum_{j=1}^{n-1}\psi \left(G/PO_{j}^{4}\right)+\sum_{i=1}^{r-1}\psi \left(G/LO_{i}\right)]. \end{array}$$

□

Now consider the case r>c of WNR (r,c,n) in which the subcase c=r−1 holds for the Theorem 1, but the other subcases do not. Furthermore, the Θ -classes behave strangely in cases where 1≤c≤r−2 , making it difficult to identify common patterns. Therefore, we discuss the case c=r−2 and other cases can be dealt with in a similar way.

**Theorem** **2.** *Let G be a wave-like nanoribbons* WNR (r,r−2,n) *, where n≥1 , and r≥3 .*

*1.*  *
$\begin{array}{l} W(G)=\frac{2}{15}(r^{5}\left(80n^{3}+25n-6\right)-r^{4}\left(80n^{3}-55n+20\right)-r^{3}\left(320n^{3}+200n-45\right)+r^{2}\left(320n^{3}+50n-70\right)+\\ r(320n^{3}-235n+351)-320n^{3}+410n-360) \end{array}$
*
*2.*  *
$\begin{array}{l} W_{e}\left(G\right)=\frac{1}{30}(r^{5}\left(720n^{3}+225n-54\right)-r^{4}\left(1200n^{3}+900n^{2}-225n+150\right)-r^{3}\left(1840n^{3}-840n^{2}+1545n-530\right)+\\ r^{2}\left(3120n^{3}+2220n^{2}+1635n-840\right)+r\left(1200n^{3}-1200n^{2}-2360n+2164\right)-2000n^{3}-1500n^{2}+2030n-1920) \end{array}$
*
*3.*  *
$\begin{array}{l} W_{ev}\left(G\right)=\frac{1}{30}(r^{5}\left(480n^{3}+150n-36\right)-r^{4}\left(640n^{3}+300n^{2}-240n+110\right)-r^{3}\left(1600n^{3}-180n^{2}+1200n-315\right)+\\ r^{2}\left(2080n^{3}+900n^{2}+720n-445\right)+r\left(1280n^{3}-360n^{2}-1240n+1656\right)-1600n^{3}-600n^{2}+1780n-1650) \end{array}$
*
*4.*  *
$\begin{array}{l} Sz_{v}\left(G\right)=\frac{1}{3}(48n^{3}r^{6}-r^{5}\left(16n^{3}-10n+3\right)-r^{4}\left(272n^{3}-38n+35\right)+r^{3}\left(64n^{3}-60n+12\right)+r^{2}(512n^{3}-362n+\\ 368)-r\left(64n^{3}-654n+606\right)-320n^{3}-268n+258) \end{array}$
*
*5.*  *
$\begin{array}{l} Sz_{e}\left(G\right)=\frac{1}{30}(1080n^{3}r^{6}-r^{5}\left(1080n^{3}+1440n^{2}-465n+63\right)-r^{4}\left(5040n^{3}-300n^{2}-1635n+720\right)+r^{3}(3560n^{3}+\\ 7140n^{2}-4375n+165)+r^{2}\left(8400n^{3}-2520n^{2}-6015n+6720\right)-r\left(3000n^{3}+7380n^{2}-15020n+10332\right)-\\ 5000n^{3}+2100n^{2}-6940n+4320) \end{array}$
*
*6.*  *
$\begin{array}{l} Sz_{ev}\left(G\right)=\frac{1}{60}(1440n^{3}r^{6}-r^{5}\left(960n^{3}+960n^{2}-460n+87\right)-r^{4}\left(7520n^{3}+120n^{2}-1500n+940\right)+r^{3}(3520n^{3}+\\ 5040n^{2}-3960n+595)+r^{2}\left(13280n^{3}-600n^{2}-8520n+8800\right)-r\left(3200n^{3}+6240n^{2}-19440n+14908\right)-\\ 8000n^{3}+1680n^{2}-8560n+6420) \end{array}$
*
*7.*  *
$PI(G)=\frac{2}{3}\left(54n^{2}r^{4}-r^{3}\left(36n^{2}+33n-4\right)-r^{2}\left(174n^{2}-12\right)+r\left(60n^{2}+93n-37\right)+150n^{2}-21n+18\right)$
*
*8.*  *
$\begin{array}{l} S(G)=\frac{2}{15}(r^{5}\left(480n^{3}+150n-36\right)-r^{4}\left(640n^{3}+120n^{2}-240n+110\right)-r^{3}\left(1600n^{3}-120n^{2}+1230n-315\right)+\\ r^{2}\left(2080n^{3}+240n^{2}+720n-445\right)+r\left(1280n^{3}-240n^{2}-1180n+1656\right)-1600n^{3}+1780n-1650) \end{array}$
*
*9.*  *
$\begin{array}{l} Gut(G)=\frac{1}{15}(r^{5}\left(1440n^{3}+450n-108\right)-r^{4}\left(2400n^{3}+720n^{2}-450n+300\right)-r^{3}(3680n^{3}-960n^{2}+3600n-\\ 1100)+r^{2}\left(6240n^{3}+960n^{2}+3330n-1545\right)+r\left(2400n^{3}-1200n^{2}-3490n+3958\right)-4000n^{3}+3850n-3660) \end{array}$
*
*10.*  *
$\begin{array}{l} Mo_{v}\left(G\right)=r^{4}\left(12n^{2}-10\right)-r^{3}\left(4n^{2}+4n-30\right)-r^{2}\left(44n^{2}+28\right)+r\left(8n^{2}+8n+24\right)+40n^{2}-20+4r(1-\\ {\left(-1\right)}^{n})\sum_{i=1}^{r-2}|3r^{2}-4ir-2r-2|+4r\left(1+{\left(-1\right)}^{n}\right)\sum_{i=1}^{r-2}\left|r^{2}-4ir-2r+2\right| \end{array}$
*
*11.*  *
$\begin{array}{l} Mo_{e}\left(G\right)=\frac{1}{6}(r^{4}\left(108n^{2}-90\right)-r^{3}\left(72n^{2}+36n-292\right)-r^{2}\left(348n^{2}-12n+306\right)+r\left(120n^{2}+60n+224\right)+\\ 300n^{2}-168)+2r\left(1-{\left(-1\right)}^{n}\right)\sum_{i=1}^{r-2}|2i-8r-12ir+9r^{2}-4|+2r\left(1+{\left(-1\right)}^{n}\right)\sum_{i=1}^{r-2}\left|2i-6r-12ir+3r^{2}+6\right| \end{array}$
*

**Proof.**  To compute the topological expression for the case c=r−2 of WNR (r,c,n) , we use the same H-type Θ -classes of Theorem 1, and the O-type Θ -classes, $PO_{i,j}^{1}$ and $PO_{j}^{4}$ do not exist. In addition, the ranges of $SO_{i}$ and $LO_{i}$ have been changed to 1≤i≤r−2 , the graph parameters of $\left\{PO_{j}^{2}:1\leq j\leq n-1\right\}$ have been changed to $|PO_{j}^{2}|=3\left(r-1\right)$ , $n_{1}\left(PO_{j}^{2}\right)=\left(r^{2}-2\right)\left(4j-1\right)$ , $m_{1}\left(PO_{j}^{2}\right)=\frac{1}{2}\left(3r^{2}\left(4j-1\right)-r\left(4j+3\right)-20j+8\right)$ and an inclusion of new Θ -class EO with parameters |EO| = 2(r−1) , $n_{1}\left(EO\right)$ = $r^{2}+2r-5$ and $m_{1}\left(EO\right)$ = $\frac{1}{2}\left(r+2\right)\left(3r-5\right)$ . Consequently, such changes are applicable to A-type Θ -classes and the computation of descriptors is performed as in Theorem 1. □

### 3.2. Degree Based Topological Descriptors

We have adopted the edge partition approach to compute some of the degree based topological descriptors for wave-like nanoribbons WNR (r,c,n) in this section.

The analytical formulas of topological descriptors for degree and degree-sum types are derived using the following equations and Table 3 and Table 4, where G=WNR(r,c,n)

$$\begin{array}{cc} \psi^{d}\left(G\right) & =d_{22}\left(G\right)\psi \left(2,2\right)+d_{23}\left(G\right)\psi \left(2,3\right)+d_{33}\left(G\right)\psi \left(3,3\right). \end{array}$$

$$\begin{array}{cc} \psi^{s}\left(G\right)= & s_{54}\left(G\right)\psi \left(5,4\right)+s_{55}\left(G\right)\psi \left(5,5\right)+s_{57}\left(G\right)\psi \left(5,7\right)+s_{58}\left(G\right)\psi \left(5,8\right)+s_{68}\left(G\right)\psi \left(6,8\right)+\\ & s_{76}\left(G\right)\psi \left(7,6\right)+s_{79}\left(G\right)\psi \left(7,9\right)+s_{88}\left(G\right)\psi \left(8,8\right)+s_{89}\left(G\right)\psi \left(8,9\right)+s_{99}\left(G\right)\psi \left(9,9\right). \end{array}$$

**Theorem** **3.** *The additive degree descriptors are given by*

*1.* 
${M_{1}}^{d}\left(G\right)=36cnr+16cn+36nr-10r-20n$
*2.* 
${M_{2}}^{d}\left(G\right)=54cnr+12cn+54nr-21r-38n+4$
*3.* 
$\begin{array}{l} {SO}^{d}\left(G\right)=2cn\left(9\sqrt{2}r-2\left(3\sqrt{2}-2\sqrt{13}\right)\right)+2\sqrt{2}n\left(9r-5\right)-\left(15\sqrt{2}-4\sqrt{13}\right)r+4(5\sqrt{2}-\\ 2\sqrt{13}) \end{array}$
*4.* 
${ABC}^{d}\left(G\right)=\frac{2}{3}\left(2cn\left(3r+3\sqrt{2}-2\right)+3n\left(2r+\sqrt{2}-2\right)+\left(3\sqrt{2}-5\right)r-3\sqrt{2}+4\right)$
*5.* 
${GA}^{d}\left(G\right)=\frac{1}{5}\left(2cn\left(15r+2\left(4\sqrt{6}-5\right)\right)+10n\left(3r-1\right)+\left(8\sqrt{6}-25\right)r-8\left(2\sqrt{6}-5\right)\right)$
*6.* 
${AZ}^{d}\left(G\right)=\frac{1}{64}\left(4374cnr+1180cn+4374nr-1597r-2326n+868\right)$
*7.* 
$\begin{array}{l} {SC}^{d}\left(G\right)=\frac{1}{30}(30\sqrt{6}cnr-20\sqrt{6}cn+48\sqrt{5}cn+30\sqrt{6}nr-30\sqrt{6}n+60n-25\sqrt{6}r+\\ 24\sqrt{5}r+20\sqrt{6}-48\sqrt{5}+60) \end{array}$
*8.* 
${ISI}^{d}\left(G\right)=\frac{1}{10}\left(90cnr+36cn+90nr-27r-50n+4\right)$
*9.* 
$F^{d}\left(G\right)=108cnr+32cn+108nr-38r-76n$
*10.* 
$H^{d}\left(G\right)=\frac{1}{15}\left(30cnr+28cn+30nr-r+2\right)$
*11.* 
${SDD}^{d}\left(G\right)=\frac{4}{3}\left(9cnr+7cn+9nr-r-3n-1\right)$

**Theorem** **4.** *The additive degree-sum descriptors are given by*

*1.* 
${M_{1}}^{s}\left(G\right)=108cnr+24cn+108nr-42r-76n+8$
*2.* 
${M_{2}}^{s}\left(G\right)=486cnr-60cn+486nr-273r-466n+124$
*3.* 
$\begin{array}{l} {SO}^{s}\left(G\right)=2n\left(27\sqrt{2}cr+2\sqrt{130}c+4\sqrt{85}c-36\sqrt{2}c+4\sqrt{145}-2\sqrt{130}+2\sqrt{89}-6\sqrt{85}+2\sqrt{74}-37\sqrt{2}+20\right)+(54\sqrt{2}n-63\sqrt{2}+\\ 4\sqrt{85}+2\sqrt{130})r+2(46\sqrt{2}+2\sqrt{41}+3\sqrt{74}-5\sqrt{85}-\sqrt{89}-\sqrt{130}-2\sqrt{145}-10) \end{array}$
*4.* 
$\begin{array}{l} {ABC}^{s}\left(G\right)=\frac{1}{630}n\left(1680cr+120\sqrt{462}c+840\sqrt{2}c-2240c-180\sqrt{462}+126\sqrt{110}+420\sqrt{30}+675\sqrt{14}+168\sqrt{2}-2660\right)+\frac{2}{63}r(84n+\\ 21\sqrt{2}+3\sqrt{462}-98)+\frac{1}{420}(255\sqrt{14}-140\sqrt{30}+168\sqrt{35}-42\sqrt{110}-100\sqrt{462}+1820-280\sqrt{2}) \end{array}$
*5.*  *
$\begin{array}{l} {GA}^{s}\left(G\right)=\frac{1}{9282}n(55692cr+11424\sqrt{42}c+13923\sqrt{7}c-74256c-17136\sqrt{42}+6188\sqrt{35}+11424\sqrt{10}-\\ 13923\sqrt{7}+21216\sqrt{3}+52416\sqrt{2}-55692)+\frac{1}{52}r\left(312n+39\sqrt{7}+32\sqrt{42}-364\right)+\frac{1}{55692}(99008\sqrt{5}-41769\sqrt{7}-\\ 34272\sqrt{10}+55692\sqrt{35}-85680\sqrt{42}-157248\sqrt{2}-63648\sqrt{3}+556920) \end{array}$
*
*6.* 
$\begin{array}{l} {AZ}^{s}\left(G\right)=\frac{1}{233744896000}(181965265539750cnr-53332033797000cn+181965265539750nr\\ -196376718849294n-117648649668375r+72697714365772) \end{array}$
*7.* 
$\begin{array}{l} {SC}^{s}\left(G\right)=\frac{1}{23205}n(23205\sqrt{2}cr+14280\sqrt{13}c-30940\sqrt{2}c+23205c+10920\sqrt{17}+6630\sqrt{14}-14280\sqrt{13}+9282\sqrt{10}+15470\sqrt{3}-\\ 54145\sqrt{2})+\frac{1}{78}r\left(78\sqrt{2}n-91\sqrt{2}+24\sqrt{13}+39\right)+\frac{1}{4641}(9282\sqrt{2}+4641\sqrt{3}-4284\sqrt{13}-663\sqrt{14}-1092\sqrt{17}+1547) \end{array}$
*8.* 
${ISI}^{s}\left(G\right)=\frac{1}{111384}\left(3007368cnr+623322cn+3007368nr-1192023r-2221914n+251063\right)$
*9.* 
$F^{s}\left(G\right)=972cnr+972nr-96cn-534r-884n+228$
*10.* 
$H^{s}\left(G\right)=\frac{1}{278460}\left(185640cnr+234430cn+185640nr+53728n+24395r+33350\right)$
*11.* 
${SDD}^{s}\left(G\right)=\frac{1}{1260}\left(15120cnr+10640cn+15120nr-2240r-3450n-191\right)$

### 3.3. Numerical Values

**Figure 5:**
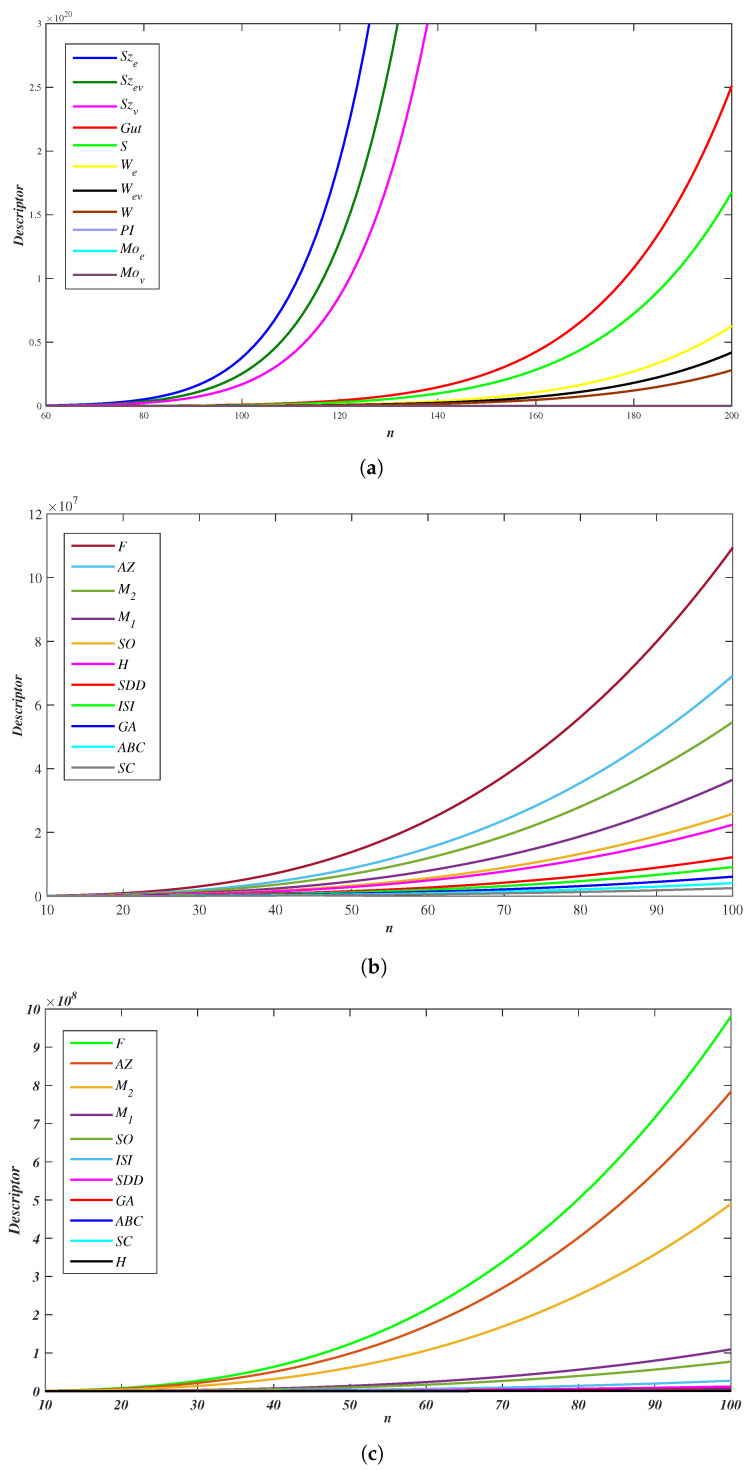
Comparative analysis of descriptors in WNR (n,n,n) (**a**) Distances (**b**) Degree (**c**) Degree-sum.

Considering the topological parameters with equal case r=c=n of WNR (r,c,n) , we present the numerical data of distance and degree-type topological descriptors for the wave-like nanoribbons in Table 5, Table 6 and Table 7. Further, we performed the plotting using MATLAB interface, as shown in Figure 5.

## 4. Applications to Stabilities and Spectroscopy of Wavy Zigzag Nanoribbons

The topological indices developed in the previous section can be applied to characterize the structures of wavy zigzag nanoribbons, and hence in QSAR predictions of the properties of these nanomaterials as a function of their structures. The adjacency and distance matrices of these structures also contain rich topological information that can be used in machine learning of stabilities and spectroscopies of these WNRs. The distance matrices of these structures can be used to generate the distance degree sequence vectors (DDSV) for each vertex of GNR, which in turn provide a technique for the vertex partitioning of GNRs. The technique is purely graph theoretical in nature and does not use any experimental reference parameters to construct the vertex partitions. Quintas and coworkers [71] have shown the use of DDSV as a means to characterize the vertices of graphs although the equivalence of DDSV is not always isomorphic with the automorphic equivalence of vertices. In this technique, we associate with each vertex $v_{i}$ in the GNR a *p*-tuple vector $\left(D_{i0},D_{i1},D_{i2},\ldots ,D_{ij},\ldots D_{ip}\right)$ where $D_{ij}$ is the number of vertices at distance *j* from $v_{i}$ . The DDSV of each vertex in any GNR can be easily generated by an algorithm that generates the distance matrix, and it needs iterations up to the maximal eccentricity of the vertices or the radius of the graph. In this algorithm, the distance matrix is generated from the adjacency matrix A where ij th element of the distance matrix is the topological distance of the shortest path from the vertex $v_{i}$ to $v_{j}$ . Thus, the DDSVs of various GNRs considered here are computed using the computer code developed previously by one of the authors [72]. The same TopoChemie-2020 package [72] was also used to validate the numerical results derived from the analytical expressions for the topological descriptors of GNRs considered here. The DDSV for each vertex is a vector of variable length with the length of DDSV of each vertex given by its eccentricity. The maximal length of the DDSV is the radius of the GNR under consideration.

Table 8 shows the machine-generated numbers of C13 NMR signals thus computed for both the composing ZNR (r,c) and WNR (r,c,n) for various values of *r*, *c*, and *n*. As the constituting units ZNR (r,c) possess $C_{2v}$ symmetry, the same symmetry is reflected in the composed wavy zigzag nanoribbons. The number of NMR signals and their intensity patterns are generated from the vertex partitioning and do not require any experimental parameters. Thus all GNRs exhibit C13 NMR signal and intensity pattern consistent with 1:1:...:1:1 ratios with the number of signals enumerated by the DDSV method as shown on Table 8. As all of the wavy zigzag nanoribbons considered here are diradicals the equivalence classes enumerated thus can be used for the machine generation of the ESR hyperfine structures of nanoribbons. Consider for example, the wavy nanoribbon WNR (3,4,5) shown in Table 8. There are 380 carbon nuclei in this nanoribbon which are partitioned into 190 classes, with each class containing two nuclei as derived from the DDSV algorithm. Thus, the ESR hyperfine structure generating function for such a case is determined by the polynomials GF shown below, where α represents spin up and β represents spin down for the C13 spin-1/2 nucleus.

GF(ESR;C13;WNR(3,4,5))=∏i=1190(αi+βi)2

**Figure 6:**
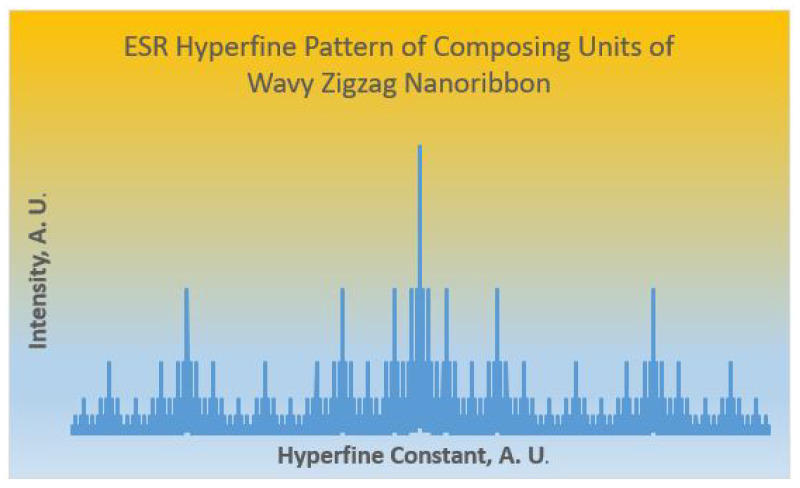
ESR hyperfine pattern arising from the composing units of wavy zigzag nanoribbon diradicals.

In the above ESR generating function, when expanded and simplified, the coefficient of each term gives the intensity of the ESR hyperfine line. This is a purely combinatorial generating function method and does not require any experimental reference standards or other experimental inputs. As the above GF generates far too many lines for illustration purposes, we show in Figure 6 the machine-generated ESR hyperfine pattern arising from the composing units of wavy zigzag nanoribbon diradicals. The ESR hyperfine patterns get composed one into the other for the wavy nanoribbons similar to the operator methods that invoke the wreath product structures considered earlier in the context of NMR spectroscopy [73]. It should be noted that the C13 - $e^{-}$ coupling depends on the Euclidean proximity of the unpaired electron and the C13 nucleus, and thus those nuclei close to the electron would have stronger interaction reducing complexity of the hyperfine pattern, although we expect the machine-generated pattern shown in Figure 6 to be a motif that will repeat or compose within the final ESR hyperfine pattern.

Thermodynamic and kinetic stabilities can be estimated for the GNRs considered using the graph spectra and other measures, such as resonance energy measures per bond which are computed from the combinatorial counts of the Kekulé structures. The parameters thus derived are shown in Table 8 for both the composing ZNR (r,c) and the nanoribbons WNR (r,c,n) . As seen from Table 8, the HOMO-LUMO gaps, which measure the kinetic stability, decrease as a *r* or *c* increases for the ZNR (r,c) (see Table 8). Indeed, the gap approaches zero quite rapidly as *r* and *c* increase. Consequently, the HOMO-LUMO gaps of WNR (r,c,n) are nearly zero for most of the ribbons. This, in turn, suggests that the wavy zigzag nanoribbons exist in triplet spin ground states, making them attractive candidates for topological spintronics.

Table 8 shows graph-theoretically derived energy measures such as delocalization energies/bond, total π -electron energy and the resonance energy per bond. The total binding energies and delocalization energies were obtained from the spectra of the graphs of WNRs. Let the spectra be denoted by the set $\left\{\lambda_{1},\lambda_{2},\cdots ,\lambda_{m}\right\}$ , where *m* is the number of vertices of WNR. For the most general case, the total π -electron energy is defined as

$$\begin{array}{c} E_{\pi }=\sum_{i=1}^{m}m_{i}\lambda_{i} \end{array}$$

where $m_{i}=0,1,2,$ is the occupancy of the orbital *i*. For an alternant WNR, the resulting graph is bipartite, and hence the total π -electron energy and delocalization energy/bond, are defined from its spectra as follows:

$$\begin{array}{cc} E_{\pi } & =\sum_{i=1}^{m/2}2\lambda_{i},\;\mathrm{where}\;\lambda_{i}\geq 0\\ E_{\mathrm{Deloc}} & =E_{\pi }-m \end{array}$$

The total π -electron energy and delocalization energies/ π -bond shown in Table 8 were computed from the above expressions. The resonance energy, on the other hand, is derived from the constant coefficient of the matching polynomials of WNR graphs, which is the number of resonance structures, or commonly called the Kekulé count, *K*. Consequently, if *K* is the coefficient of the constant term of the matching polynomial of the WNR then the resonance energy is given by Herndon’s definition [74]:

$$\begin{array}{c} \mathrm{RE}/\mathrm{bond}=\frac{1}{m}\left\{1.185\times ln\left(K\right)\right\} \end{array}$$

In Table 8, we have provided the resonance energies/bond computed with the above formula for several WNRs.

Aihara as well as Dias and coworkers [48,49,50,51,52] have carried out pioneering studies on polycyclic aromatic molecules derived from graphenes. These studies have revealed fascinating trends on aromaticity, π -electron delocalization energies, bond resonance energies, and topological resonance energies of these species [48,49,50,51,52]. It is clear that hybrid techniques through machine learning are warranted for these systems as pure ab initio quantum chemical techniques could become intractable for such nanoribbons and derivatives of graphenes. Hence, Table 8 provides some interesting trends regarding the thermodynamic stabilities and the extent of delocalization energies for these GNRs. As can be seen from Table 8 for the composing unit ZNR (r,c) , the total π -electron energy/electron and p-delocalization energies uniformly increase as the composing zigzag unit increases in dimension in either direction. The resonance energy per bond exhibits an opposite trend as a function of the dimensions, suggesting less contribution from resonance stabilization as the size increases. A similar trend is seen in Table 8 for the WNR (r,c,n) in that as the size increases in width, length or breadth the π -delocalization energy increases accompanied by a decrease in the resonance energy trend. In particular, the thermodynamic stability of the ribbon increases with the ribbon length while the kinetic stability decreases, suggesting that the longer ribbons are highly reactive yet exhibit good thermodynamic stabilities as suggested by their total π -electron energies/electron. These features suggest that the wavy zigzag nanoribbons exhibit highly reactive diradical triplet states with interesting topological features as a function of the dimensions of the ribbons, making them suitable candidates for topological spintronics, a topic of considerable interest in recent years.

A number of experimental and computational studies have been carried out earlier on WNRs of various shapes and lengths [75,76,77,78,79]. Most of these works have focused on the electronic and magnetic properties of WNRs, and in particular the HOMO-LUMO gaps, stabilities, and reactivities as functions of the length of the ribbons. Zdetsis et al. [75] have observed that the HOMO-LUMO gaps of WNRs rapidly decrease as a function of the ribbon length. This is corroborated by our results in Table 8, for the HOMO-LUMO gaps of WNRs as well as an independent study by El Abbassi et al. [76], who have observed rapid approach of metallic characters by longer WNRs. On the other hand, Jiang et al. [30] have observed enhanced reactivity of longer WNRs at the edges fully consistent with our energy gaps presented in Table 8 where zero to small HOMO-LUMO gaps suggest high kinetic instability and the reactive nature of WNRs. Moreover, Jiang and Dai [77] have shown that as the WNR increases in length, it undergoes a metamorphosis from a nonmagnetic to a magnetic ground state. This observation is fully consistent with our computed ground states of larger WNRs which exhibit diradical triplet ground states for larger nanoribbons compared to WNRs of smaller lengths which exhibit singlet ground states owing to larger HOMO-LUMO gaps for the WNRs for smaller ribbon lengths. Chopra and Maidich [78] have carried out density functional computations on WNRs for different lengths and they have noted that the binding energy increases as a function of ribbon length. As can be seen from Table 8, the column that contains the total π -electron binding energy per bond uniformly increases as a function of the ribbon length fully consistent with the DFT studies [78]. Furthermore, our computed bond resonance energies and delocalization energies support these findings. Kimouche et al. [79] have studied ultra-narrow armchair WNRs and note that the HOMO-LUMO gaps of the ribbons decrease in proportion to $1/n^{2}$ , where *n* is the ribbon length. Our computed data in Table 8 support the same trend. Furthermore, our other topological properties computed here exhibit very promising QSAR relations with other properties of such polycyclic aromatic compounds such as toxicity and biological activities as shown in a recent study on a large set of polycyclic aromatic compounds [64,80]. At present, there are no experimental data pertinent to toxicity or measurable bioactivities of WNRs. When such data become available in the future, our computed topological properties can be harnessed to develop QSAR/QSPR relations for WNRs and related nanomaterials.

Although graph theoretical techniques by themselves do not always provide accurate energetics, they facilitate hybrid techniques. In hybrid techniques, the parameters needed for the π -electron treatments, say by the PPP method, can be derived from the DFT computations on constituent ZNR or through the CASSCF/CI techniques [81] on a series of smaller zigzag strips with the active space restricted to π -electron molecular orbitals. Subsequently, machine learning techniques can be employed on a small combinatorial library of zigzag strips in order to generate the PPP parameters needed for larger GNRs. There are limitations in the CASSCF method as the number of configuration spin functions explode combinatorially as a function of the number of electrons and total number of active orbitals included in the CASSCF. The present computational limit appears to be 18 electrons distributed in all possible ways among 18 orbitals. Even then, one could carry out CASSCF with CASPT2 on several polyacenes up to tetracene (naphtacene) which contains 18 π -electrons and 18 π -orbitals. For example, a training set for such a CASPT2 could include naphthalene, antracene, phenanthrene, pyrene, triphenylene, tetracene, chrysene, benzo[a]anthracene, benco[c]anthracene, and so forth. The results of such a CASPT2 scheme on a small combinatorial library of polyacenes could provide suitable fit for the PPP integrals. On the other hand, a restricted CASSCF (RASSCF/RASPT2) technique could also be used to expand the combinatorial library. We note that a similar CASPT2 technique on ethylene was employed by Zhang et al. [82] to parameterize the 2-electron PPP integrals for polyenes. They have demonstrated that the accuracy of the results obtained by this hybrid PPP method is quite comparable to the CASPT2 method. Consequently, the graph theoretical techniques presented here provide promising pathways for the development of novel hybrid techniques to treat such large graphene-based nanomaterials.

## 5. Conclusions

We have computed the exact mathematical expressions for Wiener, Szeged-type, Schultz, and Gutman topological descriptors; additionally, a catalogue of degree-based descriptors are demonstrated for the wave-like graphene-based nanomaterial series WNR, through graph-theoretical techniques. The derived topological entities of WNR presented in this study are believed to play a predominant role in addressing the robustness and complexity of their underlying structure and thus provide significant mathematical functions that can be related to the QSAR/QSPR studies of these systems. We have also developed combinatorial tools based on distance degree sequences for the machine learning of C13 NMR and ESR hyperfine spectra of GNRs. Furthermore, our computed HOMO-LUMO gaps and various π -electron delocalization and resonance energies clearly reveal the highly reactive and yet thermodynamically stable nature of the GNRs. It is predicted that GNRs exhibit triplet ground states and are very reactive diradicals, making them ideal candidates for topological spintronics. As graphene is considered to be a highly adaptable material with significant industrial value, the GNRs are highly desirable candidates for interesting applications in spintronics, quantum physics, and information technology. Consequently, the formulated structural analysis will enhance the understanding of their geometries, stabilities, electronic and magnetic properties.

## Figures and Tables

**Table 1:** H-type Θ -classes of WNR (r,c,n) .

Θ	Range	|Θ|	$n_{1}\left(\Theta \right)$	$m_{1}\left(\Theta \right)$
$LH_{i,j}$	1≤i≤c 1≤j≤n	r+1	2r(i−2c+2j+2cj−2)+2i−4c+4cj−1	r(3i−6c+6j+6cj−7)+2i−4c−2j+4cj
$UH_{j}$	1≤j≤n	*r*	2(2j−1)(cr+c+r)	r(6j−3c+6cj−4)+4cj−2j−2c+1
$BH_{j}$	1≤j≤n−1	*r*	4j(cr+c+r)	r(6j+6cj−1)+4cj−2j

**Table 2:** O-type Θ -classes of WNR (r,c,n) .

Θ	Range	|Θ|	$n_{1}\left(\Theta \right)$	$m_{1}\left(\Theta \right)$
$SO_{i}$	1≤i≤r−1	c+i+2	$i^{2}+2i\left(c+2\right)+2c+1$	$\frac{1}{2}\left(4c+9i+6ci+3i^{2}-2\right)$
$PO_{i,j}^{1}$	1≤i≤c−r+1 1≤j≤n	r+1	$r^{2}+2r\left(i-c+2j+2cj-1\right)-2c+2i+4cj-2$	$\frac{1}{2}\left(3r^{2}+3r\left(2i-2c+4j+4cj-3\right)-4c+4i-4j+8cj-4\right)$
$PO_{j}^{2}$	1≤j≤n−1	r+c+1	$4rj\left(c+1\right)-r^{2}+2c+4cj+2$	$\frac{1}{2}\left(r\left(12j+12cj-1\right)-3r^{2}+4c-4j+8cj+4\right)$
$PO_{i,j}^{3}$	1≤i≤r−2 1≤j≤n−1	r+c+2	$2r\left(i+2j+2cj\right)-r^{2}+2c+4i+2ci+4cj+2$	$\frac{1}{2}\left(r\left(6i+12j+12cj-1\right)-3r^{2}+4c+10i-4j+6ci+8cj+2\right)$
$PO_{j}^{4}$	1≤j≤n−1	r+c+1	$r^{2}+2r\left(c+2j+2cj+1\right)+4cj-2$	$\frac{1}{2}\left(3r^{2}+3r\left(2c+4j+4cj+1\right)-2c-4j+8cj-8\right)$
$LO_{i}$	1≤i≤r−1	i+1	i(i+2)	$\frac{1}{2}\left(3i^{2}+3i-2\right)$

**Table 3:** Edge degree partition of wave-like nanoribbons.

Bond	Bond Degree	Number of Apperance in
X−Y	$d_{X}-d_{Y}$	WNR (r,c,n)
C−C	2−2	4(n+1)
	2−3	4(2cn+r−2)
	3−3	6cnr+6nr−4cn−5r−6n+4
C−H	−	2(2cn+r)

**Table 4:** Edge degree-sum partition of wave-like nanoribbons.

Bond	Bond Degree-sum	Number of Appearances in
X−Y	$s_{X}-s_{Y}$	WNR (r,c,n)
C−C	5−4	4
	5−5	4n
	5−7	2(2n+3)
	5−8	2(2n−1)
	6−8	2(2n−1)
	7−6	2(4cn+2r−6n−5)
	7−9	2(2cn−2n+r−1)
	8−8	2(2n−1)
	8−9	4(2n−1)
	9−9	6cnr+6nr−8cn−7r−14n+12
C−H	−	2(2cn+r)

**Table 5:** Distance descriptors of wave-like nanoribbons WNR (n,n,n) .

	n	1	2	3	4	5	6	7	8	9	10
ψ											
*W*		198	16,988	265,090	1,995,080	9,900,014	37,435,172	116,825,002	315,961,424	764,827,734	1,694,512,428
* $W_{e}$ *		144	23,902	447,338	3,656,560	19,008,676	74,039,578	235,829,606	647,405,812	1,585,027,224	3,543,167,190
* $W_{ev}$ *		171	20,185	344,512	2,701,379	13,719,069	52,648,697	165,987,706	452,283,443	1,101,041,295	2,450,306,985
* ${Sz}_{v}$ *		360	58,230	1,336,218	13,209,706	80,974,236	363,962,990	1,314,958,190	4,038,660,258	10,939,664,656	26,808,888,486
* ${Sz}_{e}$ *		252	81,660	2,249,918	24,170,052	155,271,436	719,090,380	2,652,165,798	8,269,315,796	22,657,699,020	56,027,704,284
* ${Sz}_{ev}$ *		306	69,067	1,734,606	17,871,135	112,137,204	511,606,727	1,867,520,568	5,779,084,127	15,743,942,926	38,756,383,875
*PI*		144	6428	57,744	279,244	961,536	2,665,804	6,354,848	13,550,044	26,514,224	48,460,476
*S*		840	85,988	1,421,788	11,009,804	55,565,776	212,481,764	668,408,452	1,818,575,052	4,422,544,728	9,834,683,940
*Gut*		889	108,760	1,906,145	15,188,508	77,966,465	301,507,160	956,059,001	2,616,774,636	6,393,217,769	14,269,710,136
* ${Mo}_{v}$ *		72	2,516	21,444	1,01,172	3,42,648	9,39,300	22,21,116	47,07,444	91,68,432	1,66,95,108
* ${Mo}_{e}$ *		84	3,300	29,324	1,41,244	4,84,620	13,40,448	31,90,580	67,96,204	1,32,89,284	2,42,76,960

**Table 6:** Degree descriptors of wave-like nanoribbons WNR (n,n,n) .

	n	1	2	3	4	5	6	7	8	9	10
$\psi^{d}$											
$M_{1}$		58	436	1350	3016	5650	9468	14,686	21,520	30,186	40,900
$M_{2}$		65	582	1879	4280	8109	13,690	21,347	31,404	44,185	60,014
SO		41.292	310.539	959.915	2142.156	4009.997	6716.172	10,413.417	15,254.467	21,392.057	28,978.922
ABC		9.152	56.446	165.720	360.975	666.211	1105.426	1702.622	2481.798	3466.955	4682.092
GA		12.919	81.353	241.464	529.252	980.716	1631.858	2518.676	3677.171	5143.342	6953.190
AZ		107.391	784.844	2455.984	5530.875	10,419.578	17,532.156	27,278.672	40,069.1875	56,313.766	76422.469
SC		6.197	35.824	103.63	224.322	412.587	683.125	1050.633	1529.808	2135.347	2881.947
ISI		14.3	107.4	333.7	747.2	1401.9	2351.8	3650.9	5353.2	7512.7	10,183.4
*F*		134	1196	3834	8696	16,430	27,684	43,106	63,344	89,046	12,0860
*H*		5.933	31.467	88.733	189.733	346.467	570.93	875.133	1271.067	1770.733	2386.133
SDD		26.67	169.34	498.67	1086.67	2005.33	3326.67	5122.67	7465.33	10,426.67	14,078.67

**Table 7:** Degree-sum descriptors of wave-like nanoribbons WNR (n,n,n) .

	n	1	2	3	4	5	6	7	8	9	10
$\psi^{s}$											
$M_{1}$		130	1164	3758	8560	16,218	27,380	42,694	62,808	88,370	120,028
$M_{2}$		297	4238	14863	35,088	67,829	116,002	182,523	270,308	382,273	521,334
SO		93.747	828.213	2666.904	6068.026	11,489.785	19,390.384	30,228.029	44,460.926	62,547.279	84,945.295
ABC		7.444	41.034	116.806	250.759	458.893	757.210	1161.709	1688.389	2353.250	3172.294
GA		12.783	81.439	241.984	530.419	982.744	1634.959	2523.063	3683.058	5150.942	6962.716
AZ		296.352	6053.189	22,252.391	53,564.827	104,661.365	180,212.873	284,890.219	423,364.271	600,305.898	820,385.968
SC		4.092	22.104	62.581	134.008	244.874	403.656	618.847	898.929	1252.389	1687.712
ISI		31.20	287.338	932.669	2129.192	4038.907	6823.815	10,645.914	15,667.207	22,049.691	29,955.368
*F*		658	8672	30,102	70,780	136,538	233,208	366,622	542,612	767,010	1,045,648
*H*		2.576	12.048	32.538	68.045	122.569	200.111	304.669	440.245	610.837	820.447
SDD		27.777	168.594	494.301	1076.896	1988.380	3300.753	5086.015	7416.166	10,363.205	13,999.134

**Table 8:** Spectral and energetic properties of GNRs.

Strutures	C13 NMR Pattern	HOMO-LUMO Gap	Total π -Electron Energy/Bond	π -Electron Delocalization Energy/Bond	Resonance Energy/Bond
ZNR (3,3)	30 signals 1:1:...:1:1 $\left(2^{30}\right)$	0.132706 β	1.46369662 β	0.4636966 β	0.1123377 β
ZNR (3,4)	38 signals 1:1:...:1:1 $\left(2^{38}\right)$	0.07387 β	1.468135457 β	0.46813546 β	0.1042822 β
ZNR (4,3)	38 signals 1:1:...:1:1 $\left(2^{38}\right)$	0.048414 β	1.47682673 β	0.47682673 β	0.10325986 β
ZNR (5,3)	46 signals 1:1:...:1:1 $\left(2^{46}\right)$	0.0184152 β	1.4854958 β	0.48549581 β	0.0953989 β
ZNR (5,4)	58 signals 1:1:...:1:1 $\left(2^{58}\right)$	0.00701 β	1.49130572 β	0.49130572 β	0.09010814 β
ZNR (9,4)	98 signals 1:1:...:1:1 $\left(2^{98}\right)$	0.000152 β	1.5096 β	0.5096296 β	0.0708 β
WNR (3,4,4)	152 signals 1:1:...:1:1 $\left(2^{152}\right)$	0.000404 β	1.4854719 β	0.4854719 β	0.10200974 β
WNR (3,4,5)	190 signals 1:1:...:1:1 $\left(2^{190}\right)$	0.00074 β	1.48667696 β	0.48667696 β	0.1018617 β
WNR (4,3,5)	190 signals 1:1:...:1:1 $\left(2^{190}\right)$	0.0 β	1.50358001 β	0.5035800 β	0.1018617 β
WNR (3,4,6)	228 signals 1:1:...:1:1 $\left(2^{228}\right)$	0.000014 β	1.4874809132 β	0.48748091 β	0.1017633 β
WNR (5,3,5)	230 signals 1:1:...:1:1 $\left(2^{230}\right)$	0.0 β	1.51395311 β	0.5139531 β	0.0913716 β

## Data Availability

Not applicable.
