# Correction: Arockiaraj et al. Topological and Spectral Properties of Wavy Zigzag Nanoribbons. *Molecules* 2023, *28*, 152

**DOI:** 10.3390/molecules31111776

**Published:** 2026-05-22

**Authors:** Micheal Arockiaraj, J. Celin Fiona, S. Ruth Julie Kavitha, Arul Jeya Shalini, Krishnan Balasubramanian

**Affiliations:** 1 Department of Mathematics, Loyola College, Chennai 600034, India; 2 Department of Mathematics, Women’s Christian College, Chennai 600006, India; 3 School of Molecular Sciences, Arizona State University, Tempe, AZ 85287-1604, USA

## Error in Main Text

In the original publication [1], there were several mistakes found in the main text.

On Page 5, in Theorem 1, there is a typo in Equation (10) which should be replaced by the following corrected expression:

$$\begin{array}{l} Mo_{v}\left(G\right)=r^{2}\left(12c^{2}n^{2}+24cn^{2}-4cn+6c+12n^{2}-4n+6\right)-2r^{4}+r(20c^{2}n^{2}+16cn^{2}-4cn-10c-4n^{2}-8)+8c^{2}n^{2}-8c^{2}-4cn^{2}-16c-4+\\ 2\left(1-{\left(-1\right)}^{n}\right)(r+c+1)\left|r^{2}-2c-2\right|+2\left(1+{\left(-1\right)}^{n}\right)\left(r+c+1\right)\left|2c-r^{2}+2\right| \end{array}$$

On Page 5, in Theorem 1, there is a typo in Equation (11) which should be replaced by the following corrected expression:

$$\begin{array}{l} Mo_{e}\left(G\right)=\frac{1}{6}(4r^{3}-18r^{4}+r^{2}(108c^{2}n^{2}+216cn^{2}-36cn+48c+108n^{2}-36n+42)+r\left(144c^{2}n^{2}+72cn^{2}-24cn-84c-72n^{2}+12n-40\right)+\\ 48c^{2}n^{2}-60c^{2}-48cn^{2}-120c+12n^{2}-60)+\left(1-{\left(-1\right)}^{n}\right)\left(r+c+1\right)\left|3r^{2}-r-5c-5\right|+\left(1+{\left(-1\right)}^{n}\right)\left(r+c+1\right)|r-3r^{2}+5c+5| \end{array}$$

On Page 10, in Table 5, last two rows incorrectly present the numerical values from the corresponding expressions. Hence, the last two rows of Table 5 should be replaced by the following corrected rows.
$Mo_{v}$722,51621,4441,01,1723,42,6489,39,30022,21,11647,07,44491,68,4321,66,95,108$Mo_{e}$843,30029,3241,41,2444,84,62013,40,44831,90,58067,96,2041,32,89,2842,42,76,960

On Page 10, in Table 6, the 8th and 11th rows incorrectly derive the numerical values from the respective expressions. Hence the 8th and 11th rows of Table 6 should be replaced by the following corrected rows.
SC6.19735.824103.63224.322412.587683.1251050.6331529.8082135.3472881.947H5.93331.46788.733189.733346.467570.93875.1331271.0671770.7332386.133

The authors wish to thank the reader for alerting and seeking clarifications. The authors state that the scientific conclusions are unaffected. This correction was approved by the Academic Editor. The original publication has also been updated.
